# Complete Genome Sequences and Characteristics of *Mycobacterium smegmatis* Phages Lucia, Cleatunium, and Reccee

**DOI:** 10.17912/micropub.biology.002377

**Published:** 2026-09-29

**Authors:** Victoria J Frost, Cadyn E Bagley, Logan A Beasley, Dylan C Lee, Kelsey G Martin, Elizabeth R Matthews, Myles A Moore, Emmie D Nightingale, Nathaniel S Pham, Emarie M Toirkens, Karen Uribe-Huizache, Kristi M Westover

**Affiliations:** 1 Biology, Winthrop University, Rock Hill, SC, USA; 2 Computer Sciences, Winthrop University, Rock Hill, SC, USA; 3 Chemistry, Winthrop University, Rock Hill, SC, USA

## Abstract

Bacteriophages Lucia, Reccee, and Cleatunium were isolated from soil beneath bushes at Winthrop University using the host bacterium *Mycobacterium smegmatis* mc ^2^ 155. Both Lucia and Reccee exhibit siphovirus morphology, with genome sizes of 54,700 bp and 70,134 bp, respectively. Cleatunium has a myovirus morphology and a genome size of 155,948 bp. Lucia can form lysogens, whereas both Reccee and Cleatunium are predicted to replicate only via the lytic cycle. Lucia is classified in the F1 subcluster, Reccee belongs to the B5 subcluster, and Cleatunium is in the C1 subcluster.

**Figure 1. Particle and plaque morphologies for Lucia, Reccee, and Cleatunium:** Transmission electron micrographs of mycobacterium phages Lucia (A), Reccee (B), and Cleatunium (C). Phage lysates were negatively stained with 1% uranyl acetate, and images were taken with a JEOL JEM-1230 TEM at 100kV acceleration voltage. Lucia (D) forms clear plaques with a turbid edge (3-5 mm, n=5). Reccee (E) forms clear plaques (1.5-2 mm, n=5), and Cleatunium (F) forms relatively large, clear plaques (4-6 mm, n=7). Table: Phage sample locations and genome assembly results

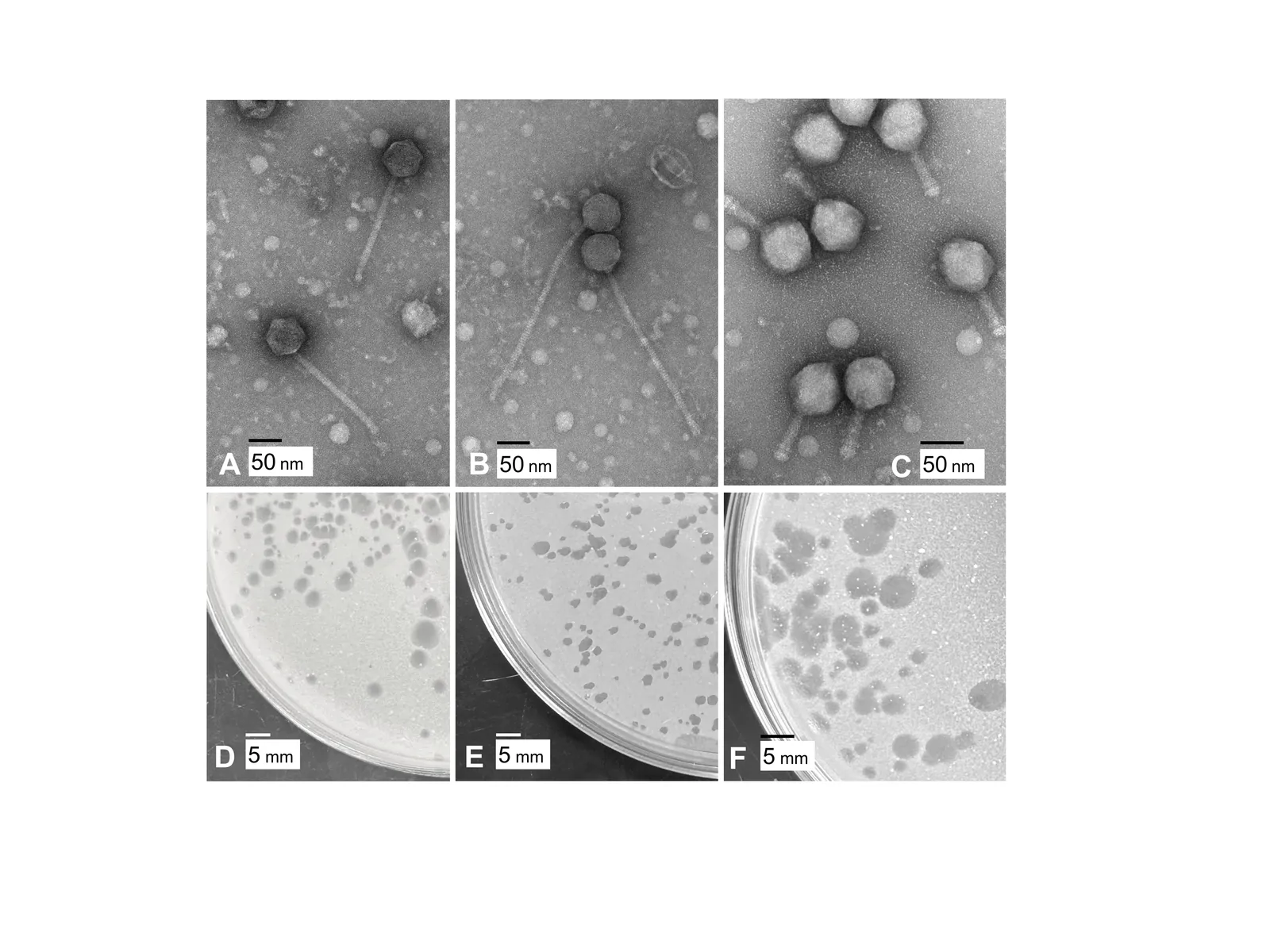



**Table:** 

Phage Details	Lucia	Reccee	Cleatunium
Sample Location (GPS)	34.94343 N, 81.0174 W	34.939958 N, 81.032637 W	34.94004 N, 81.033083 W
Sequencing Reads	774,682	377,680	416,585
Sequencing coverage (X)	1143	136	252
Genome size (bp)	54,700	70,134	155,948
Genome ends	3 prime sticky overhang (CCGATGGCAT)	Circularly permuted	Circularly permuted
GC content (%)	61.1	68.4	64.7
No. of genes	103	98	228
No. of tRNAs	0	0	32
No. of tmRNAs	0	0	1
Cluster	F1	B5	C1

## Description

Our understanding of bacteriophage biology is enhanced by large-scale phage isolation and characterization efforts (Pope et al., 2015). Such efforts advance therapeutic phage applications (Shimamura et al., 2025; Pardo-Freire et al., 2025), phage use during food processing and biocontrol (Vikram et al., 2025; Wang et al., 2026; Li et al., 2026), and the discovery of valuable phage-derived products or proteins for industrial use (Park et al., 2026; Yao et al., 2026; Gao et al., 2026). Here, we report the isolation of three phages on the bacterial host *Mycobacterium smegmatis* mc ^2^ 155.

Lucia was discovered in the soil among bushes along the banks of Winthrop University’s lake, while Reccee and Cleatunium were obtained from damp earth in the flower beds of the Little Chapel on campus (GPS coordinates are listed in Table 1). To isolate potential mycobacteriophages, each sample was shaken for 24 hours at 24 °C in buffer (10% glycerol, 1 mM CaCl _2_ ), then centrifuged at 4,000 rpm for 10 minutes. Supernatants were filtered (0.22 µm), and standard plaque assays were used to isolate, purify, and amplify all three phages (Zorawik et al., 2024) from both direct and enriched samples. The procedure for direct (unenriched) samples combined each filtrate with *M. smegmatis* , suspended in molten 7H9 agar; the mixture was overlaid onto 7H9 agar plates and incubated overnight at 37 °C. For enriched samples, filtrates were mixed with *M. smegmatis* in 7H9 medium and incubated for 4 days at 37 °C with shaking (250 rpm). Potential mycobacteriophages were collected following high-speed centrifugation (14,000 rpm) and filtration (0.22 µm) of the supernatant. The filtrate was then spotted onto an *M. smegmatis* lawn (7H9 agar plate) and incubated overnight at 37 °C. Lucia (isolated from a direct sample) created medium-sized plaques (3-5 mm, n=5) with a clear center and a turbid edge ( Figure 1D ); Reccee (isolated from an enriched sample) created plaques that were smaller (1.5-2 mm, n=5) and clear ( Figure 1E ), while Cleatunium (isolated from a direct sample) also produced clear plaques that were comparatively larger (4-6 mm, n=7) ( Figure 1F ). Lucia's ability to produce lysogens was confirmed experimentally (Moore et al., 2026). Both Lucia ( Figure 1A ) and Reccee ( Figure 1B ) exhibited siphovirus morphology with long, flexible tails (Lucia: 193-197 nm, n=4; Reccee: 327-335 nm, n=5) under transmission electron microscopy (TEM). The micrograph of Cleatunium showed a short, contractile tail (84-87 nm, n=4) characteristic of myovirus morphology ( Figure 1C ).

After two rounds of purification, each phage was amplified to generate lysates for genome extraction. Genomic DNA was extracted using the Wizard DNA Cleanup kit (Promega), and libraries prepared using the NEB Ultra II FS kit before being sequenced on an Illumina NextSeq 1000 (XLEAP-P1 kit). The resulting raw reads (100 bp) were trimmed with cutadapt v4.7 (using the option: –nextseq-trim 30) (Martin, 2011) and filtered with skewer v0.2.2 (using the options: -q 20 -Q 30 -n -l 50) (Jiang et al., 2014). The reads were then assembled with Unicycler v0.5.1 and Consed v29 (Gordon and Green, 2013; Wick et al., 2017). Table 1 describes the sequencing details and characteristics of each sequenced phage genome, including the number of putative genes, tRNAs, genome ends, and cluster assignments. Clusters were assigned based on gene content similarity (GCS), with a minimum of 35% similarity to all other sequenced phages in the Actinobacteriophage database (https://phagesdb.org/) (Russell and Hatfull, 2017; Pope et al., 2017).

Bioinformatic investigations primarily used default settings for comparison and prediction tools unless otherwise indicated. Phage genome sequences were annotated with PECAAN (v20250130) (Rinehart et al., 2016), together with Glimmer v3.02 (Delcher et al., 2007), GeneMark v4.28 (Besemer and Borodovsky, 2005), Starterator v558 (http://phages.wustl.edu/starterator/), and Phamerator (Cresawn et al., 2011), using the Actino_draft database (v657). Further analyses involved BLAST (Altschul et al., 1990) searches against the Actinobacteriophage and NCBI non-redundant databases, as well as HHpred (Söding et al., 2005) searches against the PDBmmCIF70, Pfam v37, and NCBI Conserved Domains v3.2 databases. Transmembrane domains were predicted using TOPCONS v2 (Bernsel et al., 2009), DeepTMHMM v1.0.44 (integrated into PECAAN) (Hallgren et al., 2022), and SOSUI v1.11 (Hirokawa et al., 1998). Aragorn v1.2.38 (Laslett and Canback, 2004) and tRNAscan-SE v2.0.6 (Lowe and Eddy, 1997) were used to identify transfer RNAs.

Annotation of phages Reccee and Cleatunium showed no identifiable immunity-repressor or integrase functions, suggesting the exclusive use of the lytic pathway for replication. However, Lucia’s genome revealed putative genes involved in lysogeny, including a tyrosine integrase, Cro, and an immunity repressor, and further experimental analysis demonstrated that Lucia could form lysogens (Moore et al., 2026). In all three mycobacteriophage genomes, many of the putative gene functions relate to phage structure, including capsids, tail proteins, and portal proteins. Other predicted functions include those involved in DNA genome packaging (HNH endonuclease) and replication (DNA polymerase I and/or DnaQ-like (DNA polymerase III subunit) proteins). Lucia (cluster F1) has a highly conserved predicted gene described as the mycobacteriophage mobile element 1 (MPME 1), which has been annotated across six phage clusters (Bendele et al., 2026; Cobb et al., 2026). Cleatunium (cluster C1) possesses a typical C1 cluster genome, predicted to encode numerous tRNAs (35), a tmRNA, and a putative baseplate J protein. Reccee (cluster B5) encodes a putative gene predicted to be a dpdA-like tRNA-guanine transglycosylase, thought to protect phage DNA from restriction enzymes (Hutinet et al., 2019). This gene function has been predicted in one other cluster B5 phage (Mysterious).

**Data availability**

The complete genome sequences of phages Lucia, Reccee, and Cleatunium are available in GenBank (accession no. PZ531205 , PZ531208 , and PZ789165 , respectively). The raw sequencing reads are available in the NCBI SRA under accession no. SRX34536418 , SRX34536435 , and SRX34536424 , respectively. The Actinobacteriophage sequencing BioProject accession number is PRJNA488469 .
